# Comparison of nicotine dependence between exclusive, dual, and triple tobacco users among Korean adults: A cross-sectional study

**DOI:** 10.18332/tid/219093

**Published:** 2026-05-14

**Authors:** Jung Ah Lee, Cheol Min Lee, Hong-Jun Cho

**Affiliations:** 1 Department of Family Medicine, Asan Medical Center, University of Ulsan College of Medicine, Seoul, Republic of Korea; 2 Department of Family Medicine, Seoul National University Hospital Healthcare System Gangnam Center, Seoul, Republic of Korea; 3 Department of Family Medicine, Seoul National University College of Medicine, Seoul, Republic of Korea; 4 University of Ulsan, College of Medicine, Ulsan, Republic of Korea; 5 Research Institute for Medical Sciences, Inha University Research and Business Foundation, Incheon, Republic of Korea

**Keywords:** electronic cigarettes, heated tobacco products, nicotine dependence, time-to-first tobacco use, heaviness index

## Abstract

**INTRODUCTION:**

Since the introduction of electronic cigarettes (e-cigarettes, ECs) and heated-tobacco products (heat-not-burn tobacco products, HTPs), the patterns of tobacco product use have shifted from only combustible cigarette (CC) smoking to various combinations of these three products. This study aimed to compare nicotine dependence according to exclusive, dual, and triple use among Korean adults.

**METHODS:**

This cross-sectional study used a self-reported online survey to investigate tobacco product use among Korean adults from 7 to 17 November 2022. Participants were recruited from members of an online panel via email invitations based on the 2022 Korean national population registration. Among 13288 respondents, 8000 participants (4000 men and 4000 women) were included in the final analysis. One time of EC use (approximately 15 puffs, lasting around 10 minutes) or one stick of HTP use is considered equivalent to one CC. Nicotine dependence was assessed by time-to-first tobacco use, total amount of use, and the Heaviness Index (HI).

**RESULTS:**

The prevalence of CC only users, EC only users, and HTP only users were 13.4%, 1.6%, and 2.0%, respectively. Dual users of CCs + ECs, CCs + HTPs, and ECs + HTPs were 3.4%, 4.4%, and 0.7%, respectively. Triple users were 3.2%. Compared with CC only users, the adjusted odds ratios (AORs) (95% confidence interval, CI) for high HI were 5.52 (3.18–9.57) for CC+EC users, 5.40 (3.20– 9.11) for CC+HTP users, and 4.98 (1.92–12.87) for EC+HTP users. The AOR (95% CI) for high HI was 10.95 (6.48–18.51) for triple users.

**CONCLUSIONS:**

In this study, we found that dual or triple users tended to show higher nicotine dependence than CC only users. Further studies are needed to investigate whether dual or triple use of tobacco products leads to greater nicotine dependence.

## INTRODUCTION

After the introduction of electronic cigarettes (e-cigarettes, ECs) and heated-tobacco products (heat-not-burn tobacco products, HTPs), the patterns of tobacco product use have shifted from only combustible cigarette (CC) smoking to various combinations of CC, ECs, and HTPs. In 2022, the prevalence of CCs, ECs, and HTPs in South Korea was 17.7%, 3.5%, and 5.9%, respectively^1^. Among these tobacco product users, a significant proportion were dual or triple users; 27.4% were current dual users of CC, ECs, and HTPs, and 12.4% were current triple users^2^.

Understanding nicotine dependence among these new tobacco product users is increasingly important, as their long-term health effects or influences on CC use remain unclear^3^; although previous studies reported the positive effects of EC use for smoking cessation^4,5^. Many EC users continue to smoke CCs concurrently. Even less is known about the effects of HTP use than about EC use. In addition, the measurement of nicotine dependence among new tobacco product users has not been well-established compared to CC users. However, several studies have investigated tools for measuring nicotine dependence in EC users^6,7^. Nicotine dependence among HTP users has been studied even less extensively than among EC users.

Despite the limitations in measurement, previous studies reported that dual users of CCs and ECs may have greater nicotine dependence^8,9^. HTP only users may have higher nicotine dependence than non-daily cigarette smokers^10^. Additionally, this study showed dual users of CCs and HTPs had similar nicotine dependence to daily cigarette smokers^10^. However, studies comparing nicotine dependence according to exclusive use and each combination of dual use of CCs, ECs, and HTPs are lacking. Therefore, our study aimed to compare nicotine dependence according to exclusive, dual, and triple tobacco use of CCs, ECs, and HTPs among Korean adults.

## METHODS

### Study design

We conducted a cross-sectional online survey to investigate tobacco product use among Korean adults using a sample from EMBRAIN^11^, a database company that comprised 1.6 million members. The survey was conducted from 7 to 17 November in 2022. To investigate 8000 participants, we divided the sample by quota. The number of participants for each quota was determined after matching age and provincial distributions by gender, using the 2022 national population statistics based on resident registration^12^. We sent e-mails to the randomly selected participants registered for an online panel, approximately 2–3 times the target number of participants. Each quota was filled on a first-come, first-served basis. Eligible participants were Korean adults aged ≥19 years who were members of a panel. Participants were excluded if they did not complete the questionnaire, were ineligible based on age or residence, or exceeded the pre-specified quotas. This study was approved by the Institutional Review Board of the Asan Medical Center (S2022-2183-0001) and was conducted in accordance with the Declaration of Helsinki.

### Measures

*Demographic variables and tobacco product use*

All participants completed self-reported questionnaires on age, gender, and socio-economic status. Income in KRW (1000 Korean Won about US$0.77, mean exchange rate 2022) was categorized as follows: <3000000; 3000000–4999999; 5000000–6999999; and ≥7000000. Education level was categorized as: <16 and ≥16 years. Marital status was categorized as married/cohabited, separated /divorced/widowed, and never married.

Current CC smokers were defined as those who currently smoke cigarettes ‘every day’ or ‘some days’ and have a lifetime use of ≥100 cigarettes. Those who reported smoking not at all or <100 cigarettes in their lifetime were classified as never smokers. Current EC users were defined as those who have ever used ECs in their lifetime and in the past 30 days. Never EC users were defined as those who have never used ECs in their lifetime. Current HTP users were defined as those who have used HTPs at any time in their lifetime and currently use them every day or on some days. Never HTP users were defined as those who have never used HTPs in their lifetime. The former users were defined as participants who had used CCs, ECs, or HTPs in the past but did not currently use CCs or HTPs, or had not used ECs in the past month. To minimize misunderstandings about the differences between ECs and HTPs, images and names of the ECs and HTPs (including IQOS, Glo, and Lil) were provided.

*Definition of nicotine dependence among tobacco product users*

Nicotine dependence was assessed by time to first use of tobacco products, total amount of tobacco product use, and Heaviness Index (HI)^13,14^. Among exclusive users, time to first use of tobacco products was assessed by the following question: ‘How soon after you wake up do you use your first CC (or EC or HTP) of the day?’ (≤5, 6–30, 31–60, after 60 minutes) Among dual and triple users, it was assessed using the question: ‘How soon after you wake up do you use any tobacco product?’.

For CC users, the number of cigarettes per day was assessed by the question: ‘How many cigarettes do you smoke per day?’. We used the following question for EC users: ‘How many times per day do you usually use your EC? (assume that one ‘time’ consists of around 15 puffs or lasts around 10 minutes)’. The definition of one time was based on a previous study, which reported that three well-constructed EC dependence scales demonstrated internal consistency and validity among dual users of CCs and ECs^7^. For HTP users, the number of sticks per day was assessed by the question: ‘How many sticks do you smoke per day?’. Based on time to first tobacco product use and the amount of tobacco consumption, we calculated the HI of tobacco products. The time to first cigarette was divided into four groups (≤5, 6–30, 31–60, and >60 minutes), each scored from 3 to 0. We considered one time or one stick to be the same as 1 cigarette. Thus, the amount of tobacco product consumption was calculated by adding all tobacco product use, including the number of cigarettes consumed per day for CCs, the number of times per day for ECs, and the number of sticks consumed per day for HTPs. Total tobacco product consumption was divided into four groups: ≤10, 11–20, 21–30, and >30 cigarettes (times or sticks), which were scored from 0 to 3 points, respectively. HI was calculated by adding the total scores of the two domains and ranged from 0 to 6 points. Considering all three components, participants were classified as having high nicotine dependence if they met any of the following: 1) time to first tobacco use within 30 minutes, 2) consuming more than 20 cigarettes (or times or sticks) per day, or 3) a total score of 5–6 points on the HI scale.

### Statistical analysis

Demographic characteristics of all participants were compared between men and women using chi-squared tests or Fisher’s exact tests. Among the total participants, we selected current users of tobacco products, including CCs, ECs, and HTPs, and investigated their nicotine dependence. Categorical variables are presented as percentages with 95% CI, and continuous variables expressed as mean with 95% CI. To compare nicotine dependence across tobacco product use groups, the HI was analyzed as a continuous variable using one-way analysis of variance (ANOVA), followed by Scheffé’s post hoc test for pairwise comparisons. Logistic regression for high nicotine dependence was performed after adjusting for age, gender, education level, marital status, and income, which were considered as potential confounding factors. Smoking prevalence differs markedly between men and women in Korea^15,16^, and marital status has been associated with changes in smoking behavior^17^. Similar to CC use, the use of ECs and HTPs varied according to socio-economic status, with men with a high level of education and women of low income and low level of education, being more likely to use these products^16^. As non-daily CC users would be less dependent than daily smokers on multiple dependence measures^18^, we performed logistic regression analysis after excluding non-daily CC users for sensitivity analysis. Additionally, for sensitivity analysis, multiple logistic regression was performed for high nicotine dependence in groups stratified by the three types of tobacco use: CC use, EC use, and HTP use. All statistical analyses were performed using STATA 16.1 (Stata Corporation, College Station, Texas, USA). Statistical significance was established at a two-tailed p<0.05 level.

## RESULTS

**Figure 1:**
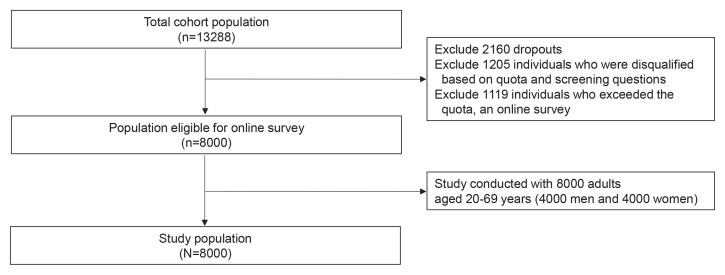
Flowchart of study participants in a cross-sectional online survey conducted in Korea, 2022

A total of 13288 participants responded and provided informed consent to participate in the survey. Of the total respondents, 2160 did not reply to any of the questions or did not complete the questionnaire. We excluded 1205 participants who were not eligible for the survey, as well as 1119 participants who exceeded the quota. Finally, 4000 men and 4000 women participated in the online survey (Figure 1).

Table 1 presents the demographic characteristics of the study population participating in the original survey. Of the total 8000 participants, the mean age was 45.0 years, and 59.9% had a college degree or higher. A total of 62.2% of participants were or had been married or cohabited. In terms of the tobacco use status of the total population, 13.4% were CC only users, 1.6% were EC only users, and 2.0% were HTP only users. Dual users of CCs + ECs, CCs + HTPs, and ECs + HTPs were 3.4%, 4.4%, and 0.7%, respectively. Triple users of CCs, ECs, and HTPs were 3.2%. Former users of any three tobacco products were 16.7% of the total population. A total of 54.5% of participants had never used any of the three tobacco products.

**Table 1:** Basic characteristics of study participants in a cross-sectional online survey conducted in Korea, 2022 (N=8000)

*Characteristics*	*Total n (%)*	*Men n (%)*	*Women n (%)*	p*
**Total**, n	8000	4000	4000	
**Age** (years), mean (SD)	44.99 (13.23)	44.88 (13.34)	45.09 (13.13)	
20–39	2818 (35.2)	1443 (36.1)	1375 (34.4)	0.165
40–59	3599 (45.0)	1793 (44.8)	1806 (45.1)	
60–69	1583 (19.8)	764 (19.1)	819 (20.5)	
**Education level** (years)				
<16	3211 (40.1)	1375 (34.4)	1835 (45.9)	<0.001
≥16	4789 (59.9)	2625 (65.6)	2164 (54.1)	
**Marital status**				
Married/cohabited	4979 (62.2)	2437 (60.9)	2542 (63.5)	<0.001
Never married	2565 (32.1)	1387 (34.7)	1178 (29.5)	
Divorced/separated/widowed	456 (5.7)	176 (4.4)	280 (7.0)	
**Income** (KRW)				
<3000000	1887 (23.6)	908 (22.7)	979 (24.5)	0.019
3000000–4999999	2541 (31.8)	1334 (33.4)	1207 (30.2)	
5000000–6999999	1781 (22.2)	875 (21.9)	906 (22.7)	
≥7000000	1791 (22.4)	883 (22.1)	908 (22.7)	
**Tobacco use status**				
Exclusive CC use	1074 (13.4)	873 (21.8)	201 (5.0)	<0.001
Exclusive EC use	131 (1.6)	78 (2.0)	53 (1.3)	
Exclusive HTP use	161 (2.0)	116 (2.9)	45 (1.1)	
Dual use of CCs and ECs	271 (3.4)	202 (5.1)	69 (1.7)	
Dual use of CCs and HTPs	351 (4.4)	285 (7.1)	66 (1.6)	
Dual use of ECs and HTPs	59 (0.7)	36 (0.9)	23 (0.6)	
Triple use of CCs, ECs, and HTPs	259 (3.2)	200 (5.0)	59 (1.5)	
Former use of any three tobacco products	1338 (16.7)	1057 (26.4)	281 (7.0)	
Never used any of the three tobacco products	4356 (54.5)	1153 (28.8)	3203 (80.1)	

* Calculated by chi-squared test or Fisher’s exact test. CC: combustible cigarette. EC: electronic cigarette. HTP: heated tobacco product. KRW: 1000 Korean Won about US$0.77, mean exchange rate 2022.

Table 2 shows the mean tobacco product consumption, the proportion of first cigarette use within 30 minutes of waking up, and the mean HI. Among exclusive users, the mean tobacco product consumption was 10.76 cigarettes/day in CC only users, 7.27 sticks/day in EC only users, and 11.60 sticks/day in HTP only users. Dual users of CCs + ECs, CCs + HTPs, and ECs + HTPs consumed 15.92, 16.01, and 12.80 cigarettes (times or sticks)/day, respectively. The total tobacco product consumption of triple users was 17.44 cigarettes (times or sticks)/day. The proportion of first tobacco use within 30 minutes of waking up was 47.2% for only CC use, 47.3% for only EC use, and 54.7% for only HTP use. Among dual tobacco product users, the percentage of first tobacco use within 30 minutes of waking up was the lowest (48.3%) in CCs + ECs users, followed by CCs + HTPs users (54.7%) and ECs + HTPs users (67.8%). Total 59.5% of triple users showed the first tobacco use within 30 minutes of waking up. The mean HI (95% CI) was 1.8 (1.71–1.89) among CC only users, 1.59 (1.35–1.83) among EC only users, and 2.01 (1.79–2.22) among HTP only users. Dual users of CCs + ECs, CCs + HTPs, and ECs + HTPs had a HI of 2.22 (2.02–2.42), 2.38 (2.22–2.55), and 2.27 (1.89–2.64), respectively. The mean HI for triple users was 2.65 (2.44–2.86), significantly higher than that of all exclusive users. Supplementary file Table 1 shows the mean HI and post hoc analysis of tobacco product use. In post hoc analysis, the HI of triple users was higher than that of any type of exclusive users, but not different from that of dual users.

**Table 2:** The amount of tobacco product use and proportion of participants reporting first tobacco use within 30 minutes of waking up among tobacco product users in a cross-sectional online survey conducted in Korea, 2022 (N=2306)

*Tobacco status*	*CC use (N=1955)*	*EC use (N=720)*	*HTP use (N=830)*	*Total tobacco product use*
	**Cigarettes (times or sticks)/day: mean (95% CI)**			
Exclusive CC use	10.76 (10.28–11.25)	NA	NA	10.76 (10.28–11.25)
Exclusive EC use	NA	7.27 (5.93–8.62)	NA	7.27 (5.93–8.62)
Exclusive HTP use	NA	NA	11.60 (10.46–12.74)	11.60 (10.46–12.74)
Dual use of CCs and ECs	8.45 (7.44–9.47)	7.47 (6.34–8.60)	NA	15.92 (14.36–17.50)
Dual use of CCs and HTPs	9.28 (8.51–10.04)	NA	6.74 (5.96–7.52)	16.01 (14.96–17.07)
Dual use of ECs and HTPs	NA	9.42 (5.45–13.40)	3.37 (1.97–4.78)	12.80 (8.65–16.95)
Triple use of CCs, ECs, and HTPs	7.83 (6.85–8.82)	6.17 (5.44–6.90)	3.44 (2.69–4.18)	17.44 (15.79–19.09)
	**First tobacco use within 30 minutes of waking up: % (95% CI)**			
Exclusive CC use	47.2 (44.2–50.2)	NA	NA	47.2 (44.2–50.2)
Exclusive EC use	NA	47.3 (38.5–56.2)	NA	47.3 (38.5–56.2)
Exclusive HTP use	NA	NA	54.7 (46.6–62.5)	54.7 (46.6–62.5)
Dual use of CCs and ECs	50.6 (44.4–56.7)	43.5 (37.6–49.7)	NA	48.3 (42.3–54.5)
Dual use of CCs and HTPs	52.1 (46.8–57.5)	NA	43.9 (38.6–49.2)	54.7 (49.3–60.0)
Dual use of ECs and HTPs	NA	64.4 (50.9–76.4)	57.6 (44.1–70.4)	67.8 (54.3–79.4)
Triple use of CCs, ECs, and HTPs	58.3 (52.0–64.4)	46.3 (40.1–52.6)	48.3 (42.0–54.5)	59.5 (53.2–65.5)
	**Heaviness Index : mean (95% CI)**			
Exclusive CC use	1.80 (1.71–1.89)	NA	NA	1.80 (1.71–1.89)
Exclusive EC use	NA	1.59 (1.35–1.83)	NA	1.59 (1.35–1.83)
Exclusive HTP use	NA	NA	2.01 (1.79–2.22)	2.01 (1.79–2.22)
Dual use of CCs and ECs	2.22 (2.02–2.42)	2.22 (2.02–2.42)	NA	2.22 (2.02–2.42)
Dual use of CCs and HTPs	2.38 (2.22–2.55)	NA	2.38 (2.22–2.55)	2.38 (2.22–2.55)
Dual use of ECs and HTPs	NA	2.27 (1.89–2.64)	2.27 (1.90–2.64)	2.27 (1.90–2.64)
Triple use of CCs, ECs, and HTPs	2.65 (2.44–2.86)	2.65 (2.44–2.86)	2.65 (2.44–2.86)	2.65 (2.44–2.86)

CC: combustible cigarette. EC: electronic cigarette. HTP: heated tobacco product. NA: not applicable.

Table 3 shows logistic regression analysis of nicotine dependence according to the type of tobacco product used. Compared with CC only users, the AOR (95% CI) for the first tobacco product uses within 30 minutes of waking up was 1.64 (1.16–2.30) for HTP only users, 1.62 (1.26–2.08) for dual users of CCs + HTPs, 2.61 (1.47–4.63) for dual users of ECs + HTPs, and 2.04 (1.53–2.73) for triple users. Any type of dual users or triple users showed a higher AOR for heavy tobacco product consumption compared with CC only users. The AOR (95% CI) was 7.93 (5.24–12.01) for dual users of CCs + ECs, 6.23 (4.20–9.25) for dual users of CCs + HTPs, and 5.32 (2.49–11.38) for dual users of ECs + HTPs. The AOR (95% CI) for triple users showed 12.96 (8.58–19.57) in heavy tobacco product consumption. The AOR (95% CI) for high HI was 5.52 (3.18–9.57) for dual users of CCs + ECs, 5.40 (3.20–9.11) for dual users of CCs + HTPs, and 4.98 (1.92–12.87) for dual users of ECs + HTPs, compared with CC only users. Also, triple users showed more than ten times higher AOR for high HI (AOR=10.95; 95% CI: 6.48–18.51) than CC only users. For sensitivity analysis, we performed multiple logistic regression after excluding non-daily CC users because non-daily CC users mostly have had lower nicotine dependence than daily CC users. Similar results were found showing that the nicotine dependence was higher among dual and triple users than exclusive users (Supplementary file Table 2). Additionally, Supplementary file Table 3 shows the results of multiple logistic regression analysis for high nicotine dependence in groups stratified by CC use, EC use, and HTP use.

**Table 3:** Logistic regression analysis of nicotine dependence according to tobacco product use in a crosssectional online survey conducted in Korea, 2022 (N=2306)

*Variables*	*n (%)*	*Univariable OR (95% CI)*	*p*	*Multivariable AOR (95% CI)*	*p*
**First tobacco use within 30 minutes of waking up**					
Exclusive CC use (ref.)	507 (47.2)	1		1	
Exclusive EC use	62 (47.3)	1.00 (0.69–1.45)	0.979	1.07 (0.74–1.56)	0.719
Exclusive HTP use	88 (54.7)	1.35 (0.97–1.88)	0.078	1.64 (1.16–2.30)	0.005
Dual use of CCs and ECs	131 (48.3)	1.05 (0.80–1.37)	0.739	1.13 (0.85–1.49)	0.408
Dual use of CCs and HTPs	192 (54.7)	1.35 (1.06–1.72)	0.015	1.62 (1.26–2.08)	<0.001
Dual use of ECs and HTPs	40 (67.8)	2.35 (1.35–4.12)	0.003	2.61 (1.47–4.63)	0.001
Triple use of CCs, ECs, and HTPs	154 (59.5)	1.64 (1.25–2.16)	<0.001	2.04 (1.53–2.73)	<0.001
**Total tobacco product uses more than 20 cigarettes (or times or sticks)/day**					
Exclusive CC use (ref.)	52 (4.8)	1		1	
Exclusive EC use	7 (5.3)	1.11 (0.49–2.50)	0.802	1.38 (0.61–3.16)	0.441
Exclusive HTP use	3 (1.9)	0.37 (0.12–1.21)	0.1	0.47 (0.14–1.52)	0.205
Dual use of CCs and ECs	69 (25.5)	6.71 (4.54–9.92)	<0.001	7.93 (5.24–12.01)	<0.001
Dual use of CCs and HTPs	74 (21.1)	5.25 (3.60–7.67)	<0.001	6.23 (4.20–9.25)	<0.001
Dual use of ECs and HTPs	10 (17.0)	4.01 (1.92–8.36)	<0.001	5.32 (2.49–11.38)	<0.001
Triple use of CCs, ECs, and HTPs	88 (34.0)	10.11 (6.92–14.78)	<0.001	12.96 (8.58–19.57)	<0.001
**High Heaviness Index** (scores 5–6)					
Exclusive CC use (ref.)	29 (2.7)	1		1	
Exclusive EC use	2 (1.4)	0.56 (0.13–2.37)	0.43	0.69 (0.16–2.96)	0.618
Exclusive HTP use	2 (1.2)	0.45 (0.11–1.92)	0.282	0.62 (0.14–2.64)	0.516
Dual use of CCs and ECs	32 (11.8)	4.82 (2.86–8.13)	<0.001	5.52 (3.18–9.57)	<0.001
Dual use of CCs and HTPs	37 (10.5)	4.25 (2.57–7.02)	<0.001	5.40 (3.20–9.11)	<0.001
Dual use of ECs and HTPs	6 (10.2)	4.08 (1.62–10.25)	0.003	4.98 (1.92–12.87)	0.001
Triple use of CCs, ECs, and HTPs	48 (18.5)	8.20 (5.05–13.30)	<0.001	10.95 (6.48–18.51)	<0.001

AOR: adjusted odds ratio. Multivariable analyses were performed after adjusting age, gender, income, marital status, and education level. CC: combustible cigarette. EC: electronic cigarette. HTP: heated tobacco product.

## DISCUSSION

In this study, we found that dual users and triple users tended to show higher nicotine dependence than CC only users. Compared with CC only users, dual users had approximately 5 times higher odds of a high HI score, and triple users had more than 10 times higher odds of a high HI score.

Consistent with this study, previous studies showed that the nicotine dependence of dual and triple users was higher than that of exclusive tobacco product users^2,19^. Using more than one type of tobacco product could lead to an increase in overall tobacco product consumption. Triple users had significantly higher odds of having a high HI score than dual users. This suggests that as the number of tobacco products used increases, the proportion of participants with high nicotine dependence also rises. Additionally, because ECs and HTPs were associated with frequent stealth use due to their easy accessibility^20,21^, they were more likely to be used by CC users with high nicotine dependence.

In our study, there was no difference in nicotine dependence between exclusive users of any type of tobacco product. In a previous study of Japanese participants, the proportion of those with time-to-first tobacco product <30 minutes was higher in HTP only users than in non-daily CC users and similar to that in CC daily users^10^. Although not directly comparable to our study, this result indicated that the nicotine dependence of HTP only users was substantial. Regarding nicotine dependence among EC users, a result from the Population Assessment of Tobacco and Health (PATH) Study showed that CC only users had a shorter time-to-first tobacco use than EC only users^22^. This study result differed from our study, showing higher nicotine dependence for CC only users. This may be because the PATH Study measured self-reported minutes of time-to-first tobacco use, whereas our study asked a multiple-choice question. The limited number of EC only users may also have influenced the results of our study.

The heaviness of smoking index is a well-known measurement tool for nicotine dependence in CC users and a good indicator of smoking cessation^14^. It consists of two items, including the time to the first cigarette after waking and the number of cigarettes smoked in a day^13,14^. Although the evidence was lacking about HI for EC or HTP users, it can be logical that the time-to-first tobacco can be applied to any tobacco product. Moreover, we assumed HI for the assessment of three tobacco products, treating one time of EC use or one stick of HTP use as equivalent to one cigarette of CC use. Because the nicotine concentration of one stick of HTP is similar to that of one cigarette of CC, we thought that one stick of HTP could be considered as one cigarette of CC^23^. Following a previous study^7^, we assumed that one time of EC consists of around 15 puffs or lasts around 10 minutes. However, because the nicotine concentration of EC varies across products, further research is needed to accurately measure nicotine intake in EC users.

### Limitations

There are several limitations in our study. First, our study is a cross-sectional study; we could not determine the causal relationship between high nicotine dependence and dual and triple tobacco users. Additionally, residual confounding may affect the observed associations, and self-reported tobacco use status may introduce misclassification of tobacco product use. However, the validity of self-reported tobacco use is generally considered acceptable^24^, and the difference from biochemically verified measures is expected to be small. Second, it is controversial whether the use of 15 puffs of ECs is equivalent to one cigarette. In particular, the biological validation has not been established to determine whether one CC can be equivalently converted into one stick of HTPs or 15 puffs of ECs. The long-term risk of ECs or HTPs has not been evaluated compared to CCs. Finally, the generalizability of the conclusions of this study may be limited by the participants’ lack of representativeness. However, we tried to reduce selection bias by matching the age, gender, and provincial distribution to reflect the national Korean population statistics from 2022. Despite these limitations, the high nicotine dependence of dual and triple tobacco product users is still a meaningful result in this study.

## CONCLUSIONS

We found that the nicotine dependence of tobacco product users tended to be greater among dual and triple users than CC only users. Considering the potentially harmful health effects^25^ and the increased difficulty of quitting tobacco products, dual and triple use represents a significant challenge to tobacco control in Korea. Therefore, it is necessary to investigate whether dual or triple use of tobacco products leads to greater nicotine dependence.

## Supplementary Material



## Data Availability

The data supporting this research are available from the authors on reasonable request.

## References

[1] Ministry of Data and Statistics. Current smoking rate. National Development Indicators. [Website in Korean]. Updated January 5, 2026. Accessed March 16, 2026. https://www.index.go.kr/unify/idx-info.do?idxCd=4237

[2] Huh Y, Min Lee C, Cho HJ. Comparison of nicotine dependence between single and multiple tobacco product users among South Korean adults. Tob Induc Dis. 2022;20(February):22. doi:10.18332/tid/145899 35291560 PMC8879064

[3] Nighbor T, Wang S, Xue Z, et al. Electronic cigarette use, related health outcomes and policy interventions in the USA: a call for research to fill evidence gaps. Tob Control. doi:10.1136/tc-2024-059019 PMC13479646 40234061

[4] Auer R, Schoeni A, Humair JP, et al. Electronic nicotine-delivery systems for smoking cessation. N Engl J Med. 2024;390(7):601-610. doi:10.1056/NEJMoa2308815 38354139

[5] Hajek P, Phillips-Waller A, Przulj D, et al. A randomized trial of e-cigarettes versus nicotine-replacement therapy. N Engl J Med. 2019;380(7):629-637. doi:10.1056/NEJMoa1808779 30699054

[6] Bold KW, Sussman S, O’Malley SS, et al. Measuring e-cigarette dependence: initial guidance. Addict Behav. 2018;79:213-218. doi:10.1016/j.addbeh.2017.11.015 29174664 PMC5807200

[7] Piper ME, Baker TB, Benowitz NL, Smith SS, Jorenby DE. E-cigarette dependence measures in dual users: reliability and relations with dependence criteria and e-cigarette cessation. Nicotine Tob Res. 2020;22(5):756-763. doi:10.1093/ntr/ntz040 30874804 PMC7368344

[8] Hwang JS, Lee CM, Lee K, Kim CY. Nicotine dependence evaluated by urinary cotinine and heaviness of smoking index among smokers, vapers, and dual users: a cross-sectional study using the Korea National Health and Nutrition Examination Survey data. Korean J Fam Med. 2021;42(3):197-203. doi:10.4082/kjfm.20.0056 34038987 PMC8164922

[9] Jones DM, Guy MC, Fairman BJ, Soule E, Eissenberg T, Fagan P. Nicotine dependence among current cigarette smokers who use e-cigarettes and cannabis. Subst Use Misuse. 2023;58(5):618-628. doi:10.1080/10826084.2023.2177961 36852436 PMC10249428

[10] Lau YK, Okawa S, Meza R, Katanoda K, Tabuchi T. Nicotine dependence of cigarette and heated tobacco users in Japan, 2019: a cross-sectional analysis of the JASTIS study. Tob Control. 2022;31:e50-e56. doi:10.1136/tobaccocontrol-2020-056237 33741741 PMC9340029

[11] Academic research service. Macromill Embrain. Accessed March 16, 2026. https://www.embrain.com/eng/research/scholarshipService

[12] Ministry of the Interior and Safety. Resident registration population and household status by administrative district. National Statistics. [Website in Korean]. Accessed March 16, 2026. https://jumin.mois.go.kr/

[13] Borland R, Yong HH, O’Connor RJ, Hyland A, Thompson ME. The reliability and predictive validity of the heaviness of smoking index and its two components: findings from the international tobacco control four country study. Nicotine Tob Res. 2010;12(Suppl 1):S45-S50. doi:10.1093/ntr/ntq038 20889480 PMC3307335

[14] Chaiton MO, Cohen JE, McDonald PW, Bondy SJ. The heaviness of smoking index as a predictor of smoking cessation in Canada. Addict Behav. 2007;32(5):1031-1042. doi:10.1016/j.addbeh.2006.07.008 16930848

[15] Jung-Choi KH, Khang YH, Cho HJ. Hidden female smokers in Asia: a comparison of self-reported with cotinine-verified smoking prevalence rates in representative national data from an Asian population. Tob Control. 2012;21(6):536-542. doi:10.1136/tobaccocontrol-2011-050012 21972062

[16] Kim JE, Lee JA, Cho HJ. Gender-based socioeconomic inequality of electronic cigarettes and heated tobacco products in Korea. J Korean Soc Res Nicotine Tob. 2024;15(3):96-106. doi:10.25055/JKSRNT.2024.15.3.96

[17] Cho HJ, Khang YH, Jun HJ, Kawachi I. Marital status and smoking in Korea: the influence of gender and age. Soc Sci Med. 2008;66(3):609-619. doi:10.1016/j.socscimed.2007.10.005 17996346

[18] Shiffman S, Ferguson SG, Dunbar MS, Scholl SM. Tobacco dependence among intermittent smokers. Nicotine Tob Res. 2012;14(11):1372-1381. doi:10.1093/ntr/nts097 22529224 PMC3482012

[19] Strong DR, Pearson J, Ehlke S, et al. Indicators of dependence for different types of tobacco product users: descriptive findings from wave 1 (2013-2014) of the Population Assessment of Tobacco and Health (PATH) study. Drug Alcohol Depend. 2017;178:257-266. doi:10.1016/j.drugalcdep.2017.05.010 28675817

[20] Lee JA, Lee C, Cho HJ. Use of heated tobacco products where their use is prohibited. Tob Control. 2023;32(2):146-152. doi:10.1136/tobaccocontrol-2020-056398 34257152

[21] Yingst JM, Lester C, Veldheer S, Allen SI, Du P, Foulds J. E-cigarette users commonly stealth vape in places where e-cigarette use is prohibited. Tob Control. 2019;28(5):493-497. doi:10.1136/tobaccocontrol-2018-054432 30097510 PMC9703983

[22] Liu G, Wasserman E, Kong L, Foulds J. A comparison of nicotine dependence among exclusive e-cigarette and cigarette users in the PATH study. Prev Med. 2017;104:86-91. doi:10.1016/j.ypmed.2017.04.001 28389330 PMC5868349

[23] Salman R, Talih S, El-Hage R, et al. Free-base and total nicotine, reactive oxygen species, and carbonyl emissions from IQOS, a heated tobacco product. Nicotine Tob Res. 2019;21(9):1285-1288. doi:10.1093/ntr/nty235 30476301 PMC6698952

[24] Li W, Liu B. Comparing cotinine and NNAL verification of self-reported smoking status among lung cancer screening eligible population from the 2007-2014 National Health and Nutrition Examination Survey (NHANES). Biomarkers. 2021;26(1):45-54. doi:10.1080/1354750X.2020.1853810 33210550

[25] Glantz SA, Nguyen N, Oliveira da Silva AL. Population-based disease odds for e-cigarettes and dual use versus cigarettes. NEJM Evid. 2024;3(3):EVIDoa2300229. doi:10.1056/EVIDoa2300229 38411454 PMC11562742

