# Supplementary material for: Comparison of nicotine dependence between exclusive, dual, and triple tobacco users among Korean adults: A cross-sectional study
Source: Tob Induc Dis. 2026 May 14;24:10.18332/tid/219093. doi: 10.18332/tid/219093 (PMC13173518; doi:10.18332/tid/219093)
Supplement: Supplementary file 1 [file TID-24-58-s1.pdf]

Supplementary Tables

Table 1. Mean heaviness index and post-hoc analysis according to tobacco product use in a cross-sectional online survey conducted in Korea, 2022 (N=2,306)

Heaviness Index	Exclusive CC use (N=1,704)		Exclusive EC use (N=131)		Exclusive HTP use (N=161)		Dual use of CCs and ECs (N=271)		Dual use of CCs and HTPs (N=351)		Dual use of ECs and HTPs (N=59)		Triple use of CCs, ECs, and HTPs (N=259)	
**Mean (SD)[a]**	**1.80 (1.47)**		**1.59 (1.41)**		**2.01 (1.37)**		**2.22 (1.70)**		**2.38 (1.57)**		**2.27 (1.46)**		**2.64 (1.70)**	
	Mean Difference[c] (95% CI)	p-value[c]	Mean Difference[c] (95% CI)	p-value[c]	Mean Difference[c] (95% CI)	p-value[c]	Mean Difference[c] (95% CI)	p-value[c]	Mean Difference[c] (95% CI)	p-value[c]	Mean Difference[c] (95% CI)	p-value[c]	Mean Difference[c] (95% CI)	p-value[c]
Exclusive CC use			-0.215 (-0.718,0.288)	0.890	0.204 (0.045,0.785)	0.871	0.415 (0.045,0.785)	0.014	0.579 (0.245,0.913)	<0.001	0.469 (-0.259,0.913)	0.514	0.846 (0.450,1.222)	<0.001
Exclusive EC use	-0.215 (-0.718,0.288)	0.890			0.418 (-0.221, 1.058)	0.494	0.630 (0.051,1.209)	0.021	0.794 (0.237, 1.351)	<0.001	0.683 (-0.169,1.536)	0.231	1.061 (0.478,1.644)	<0.001
Exclusive HTP use	0.204 (-0.256,0.663)	0.871	0.418 (-0.221,1.058)	0.494			0.212 (-0.330,0.753)	0.926	0.376 (-0.142,0.893)	0.356	0.265 (-0.563,1.093)	0.972	0.642 (0.968, 1.188)	0.008
Dual use of CCs and ECs	0.415 (0.045,0.785)	0.014	0.630 (0.051,1.209)	0.021	0.212 (-0.330, 0.753)	0.926			0.164 (0.097, 1.188)	0.941	0.053 (-0.728, 0.835)	1.000	0.431 (-0.042, 0.903)	0.106
Dual use of CCs and HTPs	0.579 (0.245, 0.913)	<0.001	0.794 (0.237,1.351)	<0.001	0.376 (-0.142,0.893)	0.356	0.164 (0.097,1.188)	0.941			-0.111 (-0.876,0.655)	1.000	0.267 (-0.179,0.712)	0.606
Dual use of ECs and HTPs	0.469 (-0.259,0.913)	0.514	0.683 (-0.169,1.536)	0.231	0.265 (-0.563,1.093)	0.972	0.053 (-0.728,0.835)	1.000	-0.111 (-0.876, 0.955)	1.000			0.377 (-0.407, 1.162)	0.819
Triple use of CCs, ECs, and HTPs	0.846 (0.450,1.222)	<0.001	1.061 (0.478,1.644)	<0.001	0.642 (0.968,1.188)	0.008	0.431 (-0.042,0.903)	0.106	0.267 (-0.179, 0.712)	0.606	0.377 (-0.407, 1.162)	0.819		

[a]The ANOVA result for the overall group was statistically significant ($p < 0.001$).

[b]Value obtained by subtracting vertical from horizontal

[c]Scheffé’s post hoc test was performed.

Abbreviations: CC, combustible cigarettes; CI, confidence intervals; EC, electronic cigarettes; HTP, heated tobacco products; SD, standard deviation

Supplementary Table 2. Logistic regression of nicotine dependence after excluding non-daily CC users in a cross-sectional online survey conducted in Korea, 2022 (N=1,729)

	N (%)	Univariable OR	p-value	Multivariable OR[a]	p-value
		OR (95% CI)		OR (95% CI)	
**First tobacco use within 30 minutes of waking up**					
Exclusive CC use	471 (55.7)	1 (Reference)		1 (Reference)	
Exclusive EC use	50 (51.6)	0.84 (0.55-1.29)	0.432	0.85 (0.55-1.31)	0.463
Exclusive HTP use	87 (56.5)	1.03 (0.73-1.46)	0.862	1.19 (0.83-1.70)	0.341
Dual use of CCs and ECs	98 (57.3)	1.07 (0.76-1.49)	0.706	1.04 (0.73-1.47)	0.835
Dual use of CCs and HTPs	152 (58.0)	1.10 (0.83-1.45)	0.517	1.27 (0.95-1.69)	0.113
Dual use of ECs and HTPs	22 (62.9)	1.34 (0.67-2.70)	0.407	1.40 (0.68-2.86)	0.357
Triple use of CCs, ECs, and HTPs	103 (96.4)	1.32 (0.94-1.86)	0.114	1.55 (1.08-2.23)	0.019
**High Heaviness Index (Score 5-6)**					
Exclusive CC use	29 (3.4)	1 (Reference)		1 (Reference)	
Exclusive EC use	1 (1.0)	0.29 (0.04-2.18)	0.230	0.36 (0.05-2.66)	0.314
Exclusive HTP use	2 (1.3)	0.37 (0.09-1.57)	0.177	0.51 (0.12-2.16)	0.358
Dual use of CCs and ECs	31 (18.1)	6.23 (3.64-10.66)	<0.001	7.05 (3.98-12.49)	<0.001
Dual use of CCs and HTPs	35 (13.4)	4.34 (2.60-7.25)	<0.001	5.62 (3.28-9.63)	<0.001
Dual use of ECs and HTPs	3 (8.6)	2.64 (0.76-9.12)	0.125	3.33 (0.94-11.78)	0.062
Triple use of CCs, ECs, and HTPs	43 (26.1)	9.92 (5.97-16.48)	<0.001	14.27 (8.12-25.08)	<0.001

[a] Multivariable analyses were performed after adjusting age, gender, income, marital status, and education

Abbreviations: CC, combustible cigarettes; CI, confidence interval; EC, electronic cigarettes; HTP, heated tobacco products; KRW, Korean won; OR, odds ratio

Supplementary Table 3. Association of tobacco product type (CC, EC, HTP) with nicotine dependence: Logistic regression results from a cross-sectional online survey conducted in Korea, 2022 (N=2,306)

	CC use (N=1,955)		EC use (N=720)		HTP use (N=830)	
	OR (95% CI)[a]	p-value	OR (95% CI)[a]	p-value	OR (95% CI)[a]	p-value
**First tobacco use within 30 minutes of waking up**						
Exclusive CC use	1 (Reference)		NA		NA	
Exclusive EC use	NA		1 (Reference)		NA	
Exclusive HTP use	NA		NA		1 (Reference)	
Dual use of CCs and ECs	1.12 (0.84-1.48)	0.436	1.21 (0.79-1.86)	0.383	NA	
Dual use of CCs and HTPs	1.61 (1.25-2.07)	<0.001	NA		1.01 (0.69-1.48)	0.965
Dual use of ECs and HTPs	NA		2.82 (1.46-5.45)	0.002	1.54 (0.81-2.94)	0.189
Triple use of CCs, ECs, and HTPs	2.03 (1.51-2.72)	<0.001	2.15 (1.38-3.36)	0.001	1.26 (0.83-1.90)	0.277
**Total tobacco product uses more than 20 cigarettes (or times or sticks)/day**						
Exclusive CC use	1 (Reference)		NA		NA	
Exclusive EC use	NA		1 (Reference)		NA	
Exclusive HTP use	NA		NA		1 (Reference)	
Dual use of CCs and ECs	8.03 (5.30-12.19)	<0.001	6.03 (2.65-13.72)	<0.001	NA	
Dual use of CCs and HTPs	6.31 (4.24-9.37)	<0.001	NA		13.38 (4.14-43.28)	<0.001
Dual use of ECs and HTPs	NA		4.15 (1.46-11.77)	0.007	12.02 (3.13-46.10)	<0.001
Triple use of CCs, ECs, and HTPs	13.19 (8.71-20.00)	<0.001	9.96 (4.38-22.67)	<0.001	27.27 (8.39-88.69)	<0.001
**High Heaviness Index (scores 5-6)**						
Exclusive CC use	1 (Reference)		NA		NA	
Exclusive EC use	NA		1 (Reference)		NA	
Exclusive HTP use	NA		NA		1 (Reference)	
Dual use of CCs and ECs	5.72 (3.29-9.95)	<0.001	8.46 (1.98-36.24)	0.004	NA	
Dual use of CCs and HTPs	5.47 (3.23-9.25)	<0.001	NA		8.62 (2.04-36.42)	0.003
Dual use of ECs and HTPs	NA		7.97 (1.53-41.44)	0.014	7.66 (1.48-39.76)	0.015
Triple use of CCs, ECs, and HTPs	11.28 (6.66-19.13)	<0.001	17.11 (4.03-72.59)	<0.001	16.54 (3.92-69.76)	<0.001

[a] Multivariable analyses were performed after adjusting age, gender, income, marital status, and education

Abbreviations: CC, combustible cigarettes; CI, confidence interval; EC, electronic cigarettes; HTP, heated tobacco products; KRW, Korean won; NA, not applicable; OR, odds ratio

© 2026 Lee J.A. et al.
